# Evaluation of the matrix-assisted laser desorption/ionization time-of-flight mass spectrometry Bruker Biotyper for identification of *Penicillium marneffei, Paecilomyces* species, *Fusarium solani, Rhizopus* species, and *Pseudallescheria boydii*

**DOI:** 10.3389/fmicb.2015.00679

**Published:** 2015-07-08

**Authors:** Ying-Sheng Chen, Yen-Hung Liu, Shih-Hua Teng, Chun-Hsing Liao, Chien-Ching Hung, Wang-Huei Sheng, Lee-Jene Teng, Po-Ren Hsueh

**Affiliations:** ^1^Division of Infectious Diseases, Department of Internal Medicine, Cardinal Tien HospitalNew Taipei City, Taiwan; ^2^Graduate Institute of Clinical Medicine, College of Medicine, National Taiwan UniversityTaipei, Taiwan; ^3^Department of Graduate Institute of Biomedical Sciences, Chang Gung UniversityTao-Yuan, Taiwan; ^4^Department of Internal Medicine, Far Eastern Memorial HospitalTaipei, Taiwan; ^5^Department of Internal Medicine, National Taiwan University Hospital, National Taiwan University College of MedicineTaipei, Taiwan; ^6^Department of Laboratory Medicine, National Taiwan University Hospital, National Taiwan University College of MedicineTaipei, Taiwan; ^7^Department and Graduate Institute of Clinical Laboratory Sciences and Medical Biotechnology, National Taiwan UniversityTaipei, Taiwan

**Keywords:** matrix-assisted laser desorption/ionization time-of-flight mass spectrometry, *Penicillium marneffei*, *Paecilomyces species*, *Fusarium solani*, *Rhizopus* species, *Pseudallescheria boydii*

## Abstract

We evaluated the performance of matrix-assisted laser desorption ionization-time of flight mass spectrometry (MALDI-TOF MS), the MALDI Bruker Biotyper system (microflex LT; Bruker Daltonik GmbH, Bremen, Germany), on the identification of 50 isolates of clinically encountered molds, including *Penicillium marneffei* (*n* = 28), *Paecilomyces* species (*n* = 12), *Fusarium solani* (*n* = 6), *Rhizopus* species (*n* = 3), and *Pseudallescheria boydii* (*n* = 1). The isolates were identified to species levels by sequence analysis of the internal transcribed spacer (ITS) regions using primers ITS1 and ITS4. None of the 28 genetically well characterized isolates of *P. marneffei* were identified as *P. marneffei* by MALDI-TOF MS, because *P. marneffei* was not present in either Bruker general library (DB 5627) or Bruker filamentous fungi library V1.0. However, the rate of accurate identification as *P. marneffei* (score value ≥ 2.000) was 85.7% based on newly created database from one *P. marneffei* strain (NTUH-3370) by MALDI Biotyper system. Sequencing analysis of these 22 non-*P. marneffei* isolates of molds revealed seven *Paecilomyces variotii*, six *F. solani*, four *Paecilomyces lilacinus*, and one each of *Paecilomyces sinensis, Rhizopus arrhizus, R. oryzae, R. microspores*, and *P. boydii*. Although all the seven *P. variotii* isolates, four of the six *F. solani*, two of the four *P. lilacinus*, and two of the three isolates of *Rhizopus* species, and the *P. boydii* isolate had concordant identification results between MALDI-TOF MS and sequencing analysis, the score values of these isolates were all of <1.700. This study indicated that the MALDI Bruker Biotyper is ineffective for identifying *P. marneffei* and other unusual molds because of the current database limitations. Therefore, it is necessary to continuously update the MALDI-TOF MS databases.

## Introduction

Although invasive aspergillosis represents the most common opportunistic mold infection, less commonly encountered molds, such as *Fusarium Paecilomyces*, and *Zygomycetes* have increasingly been reported to cause invasive mold infections (Schalk et al., 2006; Malani and Kauffman, 2007; Hsiue et al., 2010b; Bassiri-Jahromi, 2014). Infections caused by non-*Aspergillus* molds are associated with a substantially worse outcome than invasive aspergillosis (Schalk et al., 2006; Malani and Kauffman, 2007; Hsiue et al., 2010b; Bassiri-Jahromi, 2014), which is most likely attributed both to the significant immunodeficient state of these patients and the intrinsically less susceptibility of these organisms to antifungal agents (Schalk et al., 2006; Malani and Kauffman, 2007; Hsiue et al., 2010b; Bassiri-Jahromi, 2014). *Penicillium marneffei* has become a well-recognized pathogen in humans and is an important emerging public health threat (Duong, 1996; Hsueh et al., 2000; Hung et al., 2012; Lee et al., 2012). Infection caused by this organism is frequently disseminated and is progressive in nature among immunocompromised hosts, particularly among patients with impaired cell-mediated immunity and human immunodeficiency virus (HIV) infection (Duong, 1996; Hsueh et al., 2000; Hung et al., 2012; Lee et al., 2012). A history of recent travel in Southeast Asia, the southern part of China, Thailand, Vietnam, or Indonesia is common among patients with *P. marneffei* infection outside Asia (Supparatpinyo et al., 1994; Larsson et al., 2012; Hu et al., 2013). Due to the life-threatening entity of infections caused by these non-*Aspergillus* molds, prompt diagnosis and appropriate antifungal treatments are crucial (Duong, 1996; Schalk et al., 2006; Malani and Kauffman, 2007; Hsiue et al., 2010b; Bassiri-Jahromi, 2014).

In clinical microbiology laboratories, mold identification largely based on the macroscopic and microscopic observation of colonies grown on mycological media culture of or direct examination of the organisms in the histopathological sections (Hsiue et al., 2010a; Cassagne et al., 2011; Chalupová et al., 2014). Adequate phenotypic identification of molds, especially non-*Aspergillus* molds, requires highly skilled mycologists, who are seldom found in clinical mycology laboratories (Cassagne et al., 2011). Furthermore, these methods are time-consuming and sometimes unreliable (Hsiue et al., 2010a; Cassagne et al., 2011; Chalupová et al., 2014). There have been a number of molecular methods to identify non-*Aspergillus* molds from various clinical specimens or directly identify these organisms in the infected tissue, however, these detection methods are usually available only in research laboratories (Hsiue et al., 2010a; Zhang et al., 2011).

The matrix-assisted laser desorption ionization–time of flight mass spectrometry (MALDI-TOF MS), which is now widely used in clinical microbiology laboratories, can rapidly and accurately identify different species of bacteria and fungi (Murray, 2010; Alanio et al., 2011; Putignani et al., 2011; Nenoff et al., 2013; Chao et al., 2014; Ling et al., 2014). However, species identification of these non-*Aspergillus* molds, particularly *P. marneffei*, using this method has seldom been reported (Marinach-Patrice et al., 2009; Santos et al., 2009; De Carolis et al., 2012; Del Chierico et al., 2012; Barker et al., 2014; Triest et al., 2015). In this study, we evaluated the performance of MALDI-TOF MS for the identification of clinical isolates of non-*Aspergillus* molds, especially *P. marneffei* that were confirmed to the species levels by using sequencing analysis methods.

## Materials and Methods

### Fungal Isolates

Twenty-eight preserved non-duplicate isolates of *P. marneffei* were recovered from various clinical specimens from 28 patients who were treated at National Taiwan University Hospital (NTUH), a 2900-bed teaching hospital in northern Taiwan, from January 2000 through December 2012 (**Table 1**). Among these patients, 18 had AIDS and three (two in Thailand and one in Myanmar) acquired *P. marneffei* infection outside Taiwan. Clinical specimens for isolation were inoculated onto Sabouraud dextrose agar (Becton Dickinson Microbiology Systems, Sparks, MD, USA) and incubated at 25°C in ambient air. These isolates were identified as *P. marneffei* using conventional methods.

**Table 1:** Comparison of the Bruker Biotyper MALDI TOF MS system with that of molecular method in identifying 28 isolates of *Penicillium marneffei.*

No. of Isolates (year of isolation)	Country of acquisition	AIDS	Source of isolates	Identification by sequencing internal transcribed spacer (ITS) region			Identification by MALDI biotyper with preexisting database		Cluster^b^
				Species	Accession no.	Identity (%)	Species	Score value^a^	
1 (2000)	Myanmar	-	Skin	*P. marneffei*	AB363755.1	100	*Penicillium funiculosum*	1.290	II
2 (2000)	Taiwan	-	Pleural effusion	*P. marneffei*	AB363755.1	100	*Microsporum canis*	1.243	-
3 (2000)	Taiwan	-	Spine	*P. marneffei*	AB363755.1	100	*Penicillium funiculosum*	1.468	I
4 (2001)	Taiwan	+	Lung	*P. marneffei*	AB363755.1	100	*Penicillium funiculosum*	1.435	I
5 (2001)	Taiwan	+	Blood	*P. marneffei*	AB363755.1	100	*Penicillium purpurogenum*	1.078	III
6 (2001)	Taiwan	-	Blood	*P. marneffei*	AB363755.1	100	*Penicillium rugulosum*	1.365	III
7 (2001)	Taiwan	+	Middle ear fluid	*P. marneffei*	AB363755.1	100	*Penicillium funiculosum*	1.524	II
8 (2001)	Taiwan	+	Blood	*P. marneffei*	AB363755.1	100	*Penicillium funiculosum*	1.141	III
9 (2002)	Taiwan	-	Blood	*P. marneffei*	AB363755.1	100	*M. canis*	1.280	I
10 (2003)	Taiwan	+	Blood	*P. marneffei*	AB363755.1	100	*M. canis*	1.124	I
11 (2003)	Taiwan	+	Blood	*P. marneffei*	AB363755.1	100	*M. canis*	1.159	I
12 (2004)	Taiwan	+	Blood	*P. marneffei*	AB363755.1	100	*Penicillium funiculosum*	1.013	II
13 (2004)	Taiwan	+	Blood	*P. marneffei*	AB363755.1	100	*Penicillium rugulosum*	1.463	III
14 (2006)	Taiwan	+	Blood	*P. marneffei*	AB363755.1	100	*Penicillium funiculosum*	1.511	IV
15 (2006)	Taiwan	+	Blood	*P. marneffei*	AB363755.1	100	*Penicillium funiculosum*	1.244	IV
16 (2006)	Taiwan	+	Blood	*P. marneffei*	AB363755.1	100	*Penicillium funiculosum*	1.548	III
17 (2007)	Thailand	+	Blood	*P. marneffei*	AB363755.1	100	*Aspergillus candidus*	1.119	I
18 (2007)	Thailand	+	Blood	*P. marneffei*	AB363755.1	100	*Penicillium funiculosum*	1.353	II
19 (2008)	Taiwan	-	Pleural effusion	*P. marneffei*	AB363755.1	100	*Penicillium funiculosum*	1.472	I
20 (2008)	Taiwan	+	Pleural effusion	*P. marneffei*	AB363755.1	100	*Penicillium rugulosum*	1.492	III
21 (2008)	Taiwan	-	Lung	*P. marneffei*	AB363755.1	100	*Penicillium funiculosum*	1.235	II
22 (2008)	Taiwan	-	Lymph node	*P. marneffei*	AB363755.1	100	*Penicillium funiculosum*	1.401	I
23 (2010)	Taiwan	+	Lymph node	*P. marneffei*	AB363755.1	99.6	*Penicillium funiculosum*	1.448	III
24 (2010)	Taiwan	+	Skin mass	*P. marneffei*	AB363755.1	100	*Penicillium rugulosum*	1.413	III
25 (2010)	Taiwan	+	Sputum	*P. marneffei*	AB363755.1	100	*Penicillium funiculosum*	1.304	I
26 (2011)	Taiwan	+	Pleural effusion	*P. marneffei*	AB363755.1	100	*Penicillium funiculosum*	1.614	I
27 (2012)	Taiwan	-	Lung	*P. marneffei*	AB363755.1	100	*Penicillium funiculosum*	1.399	I
28 (2012)	Taiwan	-	Blood	*P. marneffei*	AB363755.1	100	*Trichophyton rubrum*	1.281	III

*^a^All were unreliable identification.*

*^b^Principal component analysis (PCA) dendrogram generated by MALDI Biotyper mass spectra for 28 genetically well characterized isolates of *Penicilliun marneffei*. The dendrogram of the 28 isolates shows four cluster groups (I, II, III, and IV) with a default critical distance level of 850.*

The other 22 isolates of non- *P. marneffei* isolates of molds were recovered from various clinical specimens of 22 patients who were treated at the hospital from 2010 to 2012 (Hsiue et al., 2010b). Among these isolates, six were initially identified by phenotypic methods as *F. solani*, four were *Paecilomyces lilacinus*, five were *Paecilomyces variotii*, three were *Rhizopus* sp., two were *Talaromyces spectabilis*, and one each of *Paecilomyces sinensis* and *Pseudallescheria boydii*.

### Identification by Gene Sequencing Analysis

The isolates were further identified by sequence comparison analysis of the internal transcribed spacer (ITS) regions using primers ITS1 and ITS4 as previously described (Hsiue et al., 2010a,b).

### Identification by MALDI-TOF MS

For performance of MALDI-TOF MS system, the MALDI Biotyper system (microflex LT; Bruker Daltonik GmbH, Bremen, Germany) was used (Chao et al., 2014). A loopful of mycelial colonies from Sabouraud dextrose agar medium was transferred to into a 1.5-ml microtube containing 300 μl of pure water and then added 900 μl of pure ethanol. The suspension was pelleted after centrifugation at 12,000 rpm for 2 min. The supernatant was discarded and 1 ml of 75% ethanol was added, centrifuged after 12,000 rpm for 2 min, and the supernatant was discarded. The pellet was dried for 1 h. The sample was then subjected to a standard ethanol–formic acid extraction procedure. A volume of 1.0 μl of the supernatant was applied to a 96-spot polished steel target (Bruker Daltonik GmbH, Bremen, Germany) plate and dried. A saturated solution of 1.0 μl of MALDI matrix (HCCA; Bruker Daltonik) was applied to each sample and dried. Measurements were performed with Bruker Microflex^TM^ LT MALDI-TOF MS (Bruker Daltonik GmbH) using FlexControl^TM^ software with CompassFlex Series version 1.3 software and a 60 Hz nitrogen laser (337 nm wavelength). Spectra were collected in the linear positive mode in a mass range covering 1,960–20,132 m/z. Spectra ranging from the mass-to-charge ratio (m/z) 2,000–20,000 were analyzed using MALDI Biotyper automation control and the Bruker Biotyper 3.1 software and Bruker general library (DB 5627) and Bruker filamentous fungi library V1.0. (Bruker Daltonics). Identification scores of ≥2.000 indicated species-level identification, scores of 1.700–1.999 indicated genus-level identification, and scores of <1.700 indicated no identification (Chao et al., 2014). The criteria of identification using standard cut-off value of 2.0 and a lowered cut-off value of 1.4 were also evaluated (Triest et al., 2015).

The principal component analysis (PCA) dendrogram generated by MALDI Biotyper mass spectra obtained from the MALDI Biotyper data of the 28 *P. marneffei* isolates was also performed (Chen et al., 2014).

## Results

### Fungal Isolates

After 3 days of incubation, the *P. marneffei* isolates grew as mold, with a soluble red pigment diffusing into the agar. The reverse side was red or pink. When seen through a microscope, the isolates had typical features of *Penicillium* species. Colonies were sub-cultured onto brain-heart infusion agar slants (Becton Dickinson Microbiology Systems) and were incubated at 37°C for 3 days to yield the yeast phase. *P. marneffei* yeast cells appeared tubular or oblong, and some had a cross-septum (Hsueh et al., 2000). All the 28 isolates were confirmed as *P. marneffei* (identity 99.6–100%) with accession number of AB363755.1 (**Table 1**).

Among these 22 non-*P. marneffei* isolates of molds, using sequencing analysis, seven were *P. variotii*, six were confirmed as *F. solani*, four were *P. lilacinus*, and one each of *P. sinensis, Rhizopus arrhizus, R. oryzae, R. microspores*, and *P. boydii* (**Table 2**).

**Table 2:** Comparison of identification results by the MALDI Biotyper system with those of molecular method in identifying 22 non-*P. marneffei* isolates of molds.

Species identified by ITS sequencing analysis			Species identification by MALDI Biotyper with preexisting database	
Isolate no. (source)	Identity (%)	Accession number	MALDI-TOF results (best match)	Score values
***Fusarium solani* (*n* = 6)**			
1. (Eye)	100	EU314957	*F. solani*	1.622
2. (Tissue)	99	FJ426390	*M. persicolor*	1.046
3. (Eye)	98	AM412600	*F. solani*	1.392
4. (Eye)	98	AM412600	*F. solani*	1.646
5. (Urine)	98	EU314965	*F. solani*	1.186
6. (Skin pus)	99	EU314957	*F. solani*	2.01
***Paecilomyces lilacinus* (*n* = 4)**		
1. (Eye)	99	EU306174	*P. lilacinus*	1.061
2. (Tissue)	99	EU306174	*A. ochraceus*	1.004
3. (Eye)	99	AY213668	*M. gypseum*	0.98
4. (Tissue)	99	AY213668	*P. lilacinus*	0.99
***Paecilomyces variotii* (*n* = 7)**		
1. (Bronchial washing)	98	AY373941	*P. varioti*	1.514
2. (Tissue)	98	EU272527	*P. varioti*	1.251
3. (Sputum)	98	AY904061	*P. varioti*	1.651
4. (Blood)	98	AY373941	*P. varioti*	1.49
5. (Nail biopsy)	98	AY373941	*P. varioti*	1.423
6. (Bronchial washing)	99	EU037066	*P. varioti*	1.112
7. (Necrotic tissue)	100	EU037066	*P. varioti*	1.317
***Paecilomyces sinensis* (*n* = 1)**		
1. (Blood)	99	EU272527	*F. culmorum*	1.14
***Rhizopus arrhizus* (*n* = 1)**		
1. (Skin pus)	99	AF543520	*Rhizopus oryzae*	1.395
***Rhizopus oryzae* (*n* = 1)**		
1. (Bronchial washing)	99	AB109758	*R. oryzae*	1.231
***Rhizopus microspores* (*n* = 1)**		
1. (Pleural effusion)	100	EF151442	*R. microsporus*	1.562
***Pseudallescheria boydii* (*n* = 1)**		
1. (Sputum)	99	EF639871	*P. boydii*	1.163

### Identification by MALDI-TOF for *P. marneffei*

None of the 28 isolates were identified as *P. marneffei* by MALDI biotyper system, because *P. marneffei* was not present in either Bruker general library (DB 5627) or Bruker filamentous fungi library V1.0 (**Table 1**). There are also no reliable identification results to genus level for all the 28 genetically well characterized *P. marneffei* isolates.

**FIGURE 1:**
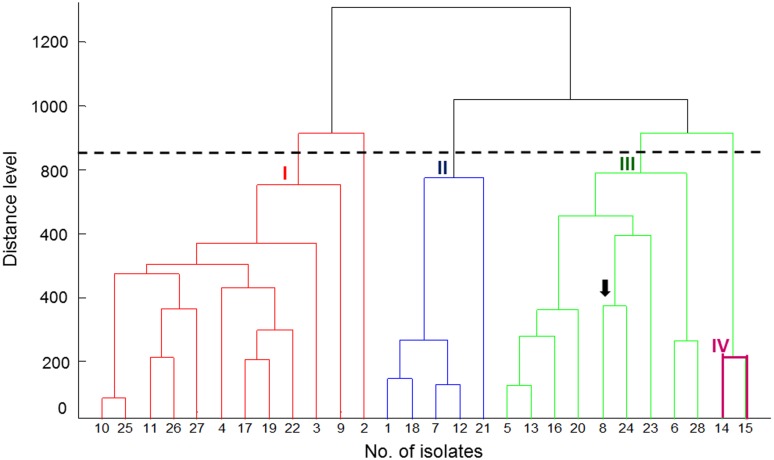
**Principal component analysis (PCA) dendrogram generated by MALDI Biotyper mass spectra for 28 genetically well characterized isolates of *Penicilliun marneffei*.** The arrow indicates the NTUH-3370 isolate. The dendrogram obtained from the MALDI Biotyper data of 25 isolates shows four cluster groups (I, II, III, and IV) with a default critical distance level of 850.

The PCA dendrogram generated by MALDI Biotyper mass spectra obtained from the MALDI Biotyper data of the 28 *P. marneffei* isolates is shown in **Figure 1**. Four cluster groups (I, II, III, and IV) with a default critical distance level of 850 were identified in 27 of the 28 isolates (**Figure 1**; **Table 1**).

### Database Creation by MALDI-TOF MS for *P. marneffei*

Because *P. marneffei* was not listed in MALDI Biotyper database, four isolates, NTUH-1124 (Isolate 4), NTUH-3370 (Isolate 8), NTUH-7204 (Isolate 12), and NTUH-9736 (Isolate 15) randomly selected from each cluster (I, III, II, and IV, respectively) of the 28 isolates of *P. marneffei* were subjected for database creation for *P. marneffei*. The eluent of each of the four well-prepared isolates was applied for eight spots on MALDI target. Each spot was performed data acquisition for four times. A total of 128 spectra were generated. After evaluation by signal resolution and mass accuracy of the 128 spectra, 27–31 of the 32 spectra from each of the four isolates (27 spectra in NTUH-1124, 29 in NTUH-3370, 28 in NTUH-7204, and 31 in NTUH-9736) were selected for MSP (Main Spectra Projection; database entrance) creation (**Table 2**). The MSP can be created by MALDI Biotyper (Bruker Daltonics) software. We then aimed at validate whether the database generated by the four strains could be used for identification of *P. marneffei*. The database was blindly tested with the 28 *P. marneffei* isolates.

### Verification of MALDI-TOF New Database of *P. marneffei*

**FIGURE 2:**
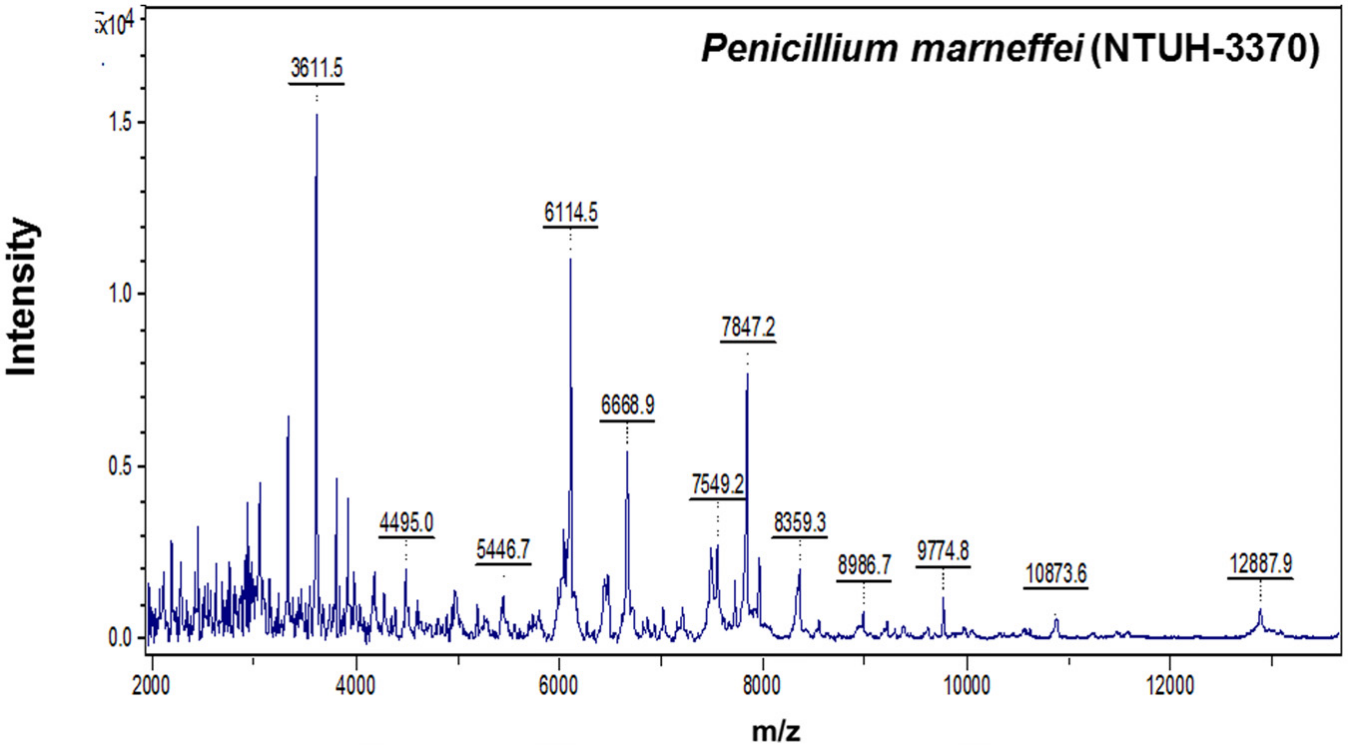
**Spectra of *Penicilliun marneffei* (NTUH-3370) generated by the MALDI-TOF Bruker Biotyper.** The absolute intensities of the ions are shown on the *y* axis, and the masses (*m/z*) of the ions are shown on the *x* axis. The *m/z* values represent the mass-to-charge ratio.

The best identification score values was found according database created by NTUH-3370: 23 (82.1%) were identified as *P. marneffei* with identification score values of ≥2.000 (2.050–2.289) and the remaining five isolates (17.9%) with score values of 1.809–1.986 (**Table 3**). Twenty, 22, and 13 isolates were identified as *P. marneffei* with score values of ≥2.000 according to MSP made by NTUH-1124, NTUH-7204, and NTUH-9736, respectively. All except one (isolates NTUH-3937 by MSP made by NTUH-7204, score value of 1.525) the remaining isolates possessed identification score values of >1.700 based on MSP made by NTUH-1124, NTUH-7204, and NTUH-9736 (**Table 3**). These characteristic MALDI Biotyper spectra of NTUH-3370 isolate are shown in **Figure 2**.

**Table 3:** Identification results of the 28 isolates of *P. marneffei* by MALDI Biotyper based on four newly created databases for *P. marneffei.*

No. of Isolates (strain designation)	Identification and score values by MALDI Biotyper with newly created database by indicated strains (designation of cluster)				
	Species	Score values^a^			
		NTUH-3370	NTUH-1124	NTUH-7204	NTUH-9736
1. (NTUH-8873)	*P. marneffei*	**1.986**	**1.844**	2.015	**1.900**
2. (NTUH-4566)	*P. marneffei*	2.050	2.029	2.011	**1.931**
3. (NTUH-4779)	*P. marneffei*	2.240	2.157	2.233	2.161
4. (NTUH-1124)	*P. marneffei*	2.464	2.790	2.545	2.313
5. (NTUH-2875)	*P. marneffei*	2.250	2.238	2.147	**1.986**
6. (NTUH-1286)	*P. marneffei*	2.162	2.113	2.266	2.163
7. (NTUH-1710)	*P. marneffei*	2.113	**1.941**	**1.995**	**1.897**
8. (NTUH-3370)	*P. marneffei*	2.770	2.481	2.491	2.265
9. (NTUH-1312)	*P. marneffei*	2.183	2.179	2.271	2.010
10. (NTUH-3937)	*P. marneffei*	**1.809**	**1.857**	1.525	**1.821**
11. (NTUH-6735)	*P. marneffei*	2.076	2.139	2.146	**1.999**
12. (NTUH-7204)	*P. marneffei*	2.559	2.467	2.831	2.451
13. (NTUH-9258)	*P. marneffei*	2.090	**1.963**	2.092	**1.954**
14. (NTUH-0594)	*P. marneffei*	2.100	2.046	2.132	2.046
15. (NTUH-9736)	*P. marneffei*	2.339	2.358	2.442	2.791
16. (NTUH-7483)	*P. marneffei*	2.289	2.089	2.114	**1.918**
17. (NTUH-7342)	*P. marneffei*	**1.860**	**1.982**	1.995	**1.961**
18. (NTUH-7732)	*P. marneffei*	2.224	2.209	2.191	2.006
19. (NTUH-1294)	*P. marneffei*	**1.964**	2.060	2.045	2.059
20. (NTUH-2810)	*P. marneffei*	2.066	**1.940**	2.077	**1.987**
21. (NTUH-0524)	*P. marneffei*	2.016	**1.970**	2.028	**1.914**
22. (NTUH-3286)	*P. marneffei*	**1.880**	**1.975**	**1.930**	**1.942**
23. (NTUH-7870)	*P. marneffei*	2.233	2.004	2.000	**1.977**
24. (NTUH-5723)	*P. marneffei*	2.268	2.127	2.143	2.123
25. (NTUH-1440)	*P. marneffei*	2.061	2.057	**1.903**	**1.909**
26. (NTUH-1934)	*P. marneffei*	2.064	2.056	2.021	2.086
27. (NTUH-0361)	*P. marneffei*	2.083	2.045	**1.996**	**1.834**
28. (NTUH-5587)	*P. marneffei*	2.102	2.116	2.247	2.186

*^a^Score values of 1.700–1.999 are shown in boldface and that of <1.700 is underlined.*

### Identification by MALDI-TOF for non-*P. marneffei* Isolates

**FIGURE 3:**
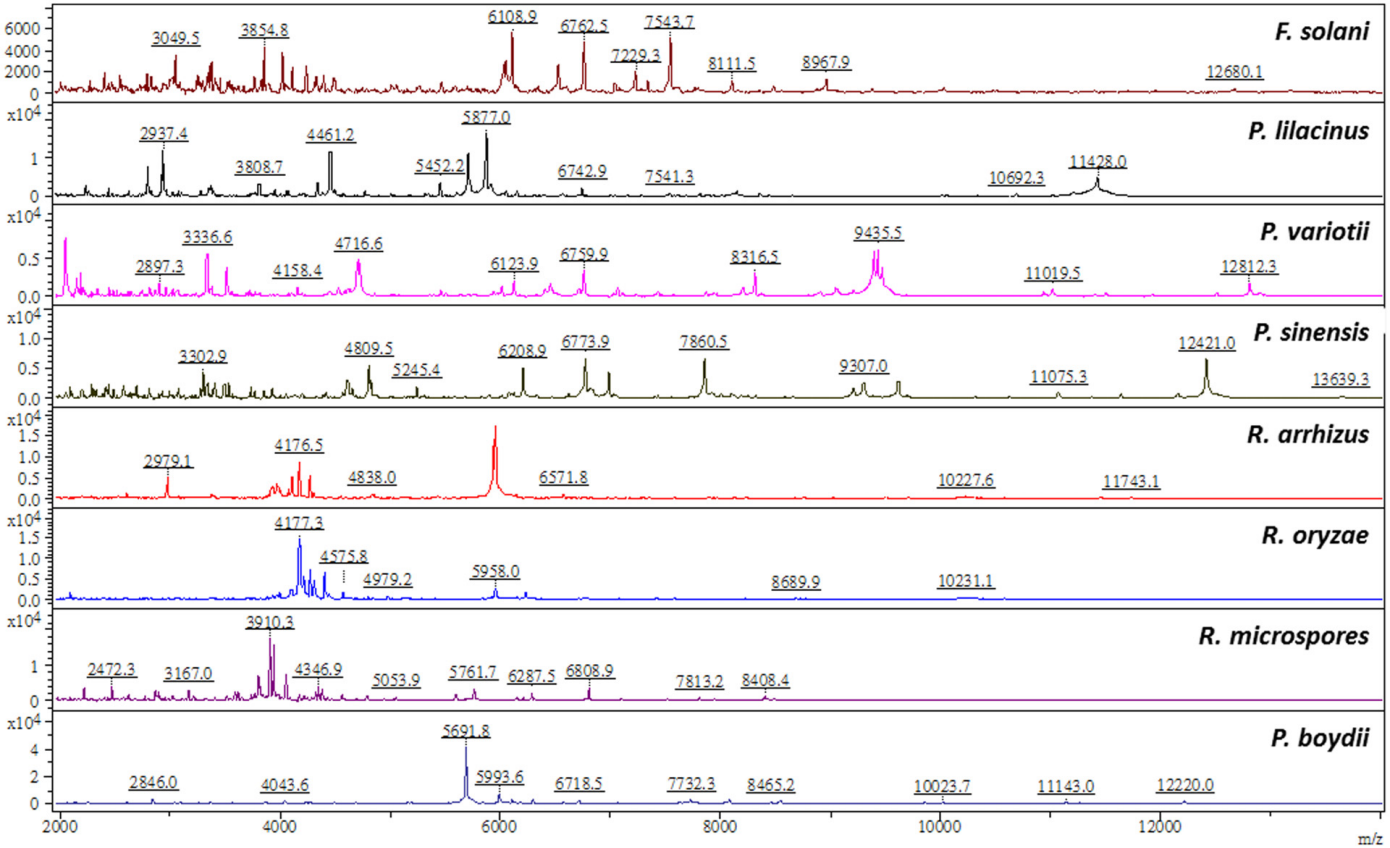
**Spectra of *Paecilomyces variotii, Fusarium solani, Paecilomyces lilacinus, Paecilomyces sinensis, Rhizopus arrhizus, Rhizopus oryzae, Rhizopus microspores*, and *Pseudallescheria boydii* generated by the MALDI-TOF Bruker Biotyper.** The absolute intensities of the ions are shown on the *y* axis, and the masses (*m/z*) of the ions are shown on the *x* axis. The *m/z* values represent the mass-to-charge ratio.

Among these 22 non-*P. marneffei* isolates of molds, only one isolate of *F. solani* could be correctly identified by MALDI-TOF MS with score value of ≥2.0 (**Table 2**). Although all the seven *P. variotii* isolates, four of the six *F. solani*, two of the four *P. lilacinus*, and two of the three isolates of *Rhizopus* species, and the *P. boydii* isolate had concordant identification results between MALDI-TOF MS and sequencing analysis, all the score values of these isolates were of <1.700 (**Table 2**). Four of the seven *P. variotii* isolates, three of the six *F. solani*, the *R. microspores* isolate, and none of other molds tested could be correctly identified with lowering the cut-off value of 1.4. These characteristic MALDI Biotyper spectra of non-*P. marneffei* isolates are shown in **Figure 3**.

## Discussion

MALDI-TOF MS is a newly developed diagnostic tool that is increasingly being employed to rapidly and accurately identify clinical relevant pathogenic yeasts and molds, including *Aspergillus, Fusarium, Zygomyces, Paecilomyces*, dematophytes, and other molds (16–27, Marinach-Patrice et al., 2009; Santos et al., 2009; Murray, 2010; Alanio et al., 2011; Putignani et al., 2011; De Carolis et al., 2012; Del Chierico et al., 2012; Nenoff et al., 2013; Barker et al., 2014; Chao et al., 2014; Ling et al., 2014; Triest et al., 2015). However, the performance of MALDI-TOF for identification of unusual or several endemic molds depends on the availability of the mold species in the database.

A study conducted by De Carolis et al. (2012) they evaluated the preexisting database of MALDI-TOF MS (MALDI Biotyper software 2.0) using 103 clinical mold isolates belonging to the 55 most clinically relevant species of *Aspergillus* (33 species), *Fusarium* (12 species), and Mucorales (10 species). Multilocus sequence analysis was used as a reference method for species designation. Excluding nine isolates that belong to the fungal species not included in our reference database, 91 (96.8%) of 94 isolates were identified by MALDI-TOF MS to the species level, in agreement with the results of the reference method and the three isolates were identified to the genus level. Recently, Triest et al. (2015) evaluated the performance of MALDI-TOF MS (MALDI Biotyper) for 268 genetically documented (multilocus sequence analysis) *Fusarium* species (19 species) and found that 82,8% of the identifications were correct up to species level according to well-defined identification criteria of score value of 2,0 using MALDI Biotyper 3.0 software (Bruker Daltonics). This success ratio could be increased up to 91% by lowering the cut-off value of 1.4 for acceptance of identification. However, among the 27 *F. solani* isolates studied, about half of the isolates were not corrected identified to species level (score values <2.0) and one-fourth of the isolates with score values <1.4. The low performance of identification of *F. solani* by MALDI-TOF MS was also noted in our study. Barker et al. (2014) evaluated the performance of MALDI-TOF MS for 77 genetically confirmed isolates of *Paecilomyces* species, the agreement was 92.2% between the molecular and proteomic methods only after supplementation of the MALDI-TOF MS database with type strains. They also indicated that *P. variotii-*like organisms required multilocus DNA interrogations for differentiation and accounted for all of the fungi whose identification was missed by MALDI-TOF MS. In a routine clinical laboratory activity (Cassagne et al., 2011), MALDI-TOF MS-based approach correctly identified 87% (154/177) of the mold isolates analyzed. The failure of identification (12%, 21/177) was found among those not represented in the reference library. In this study, one of the three *R. oryzae* isolates was misidentified as *Mucor circinelloides*.

The performance of identification of many mold genera such as *Aspergillus, Fusarium, Penicillium, Hypocrea/Trichoderma*, and some phytopathogens by MALDI-TOF MS was reviewed comprehensively by Chalupová et al. (2014). However, *P. malneffei* species have not been evaluated in these studies. In this study, using newly created database by MALDI Biotyper for NTUH-3370 strain, 85.7% of the 28 genetically well-documented *P. marneffei* isolates could be identified accurately to species-level. The remaining five isolates were also identified as *P. marneffei* although score values were >1.800 to <2.000.

## Summary

Our findings show that new database created for *P. marneffei* MALDI Biotyper could be useful and accurately identify *P. marneffei* isolates, although the number of isolates used in the study was small and only collected mainly from Taiwan. This technique could be incorporated into the work flow of the clinical mycology laboratory for rapid and accurate identification of *P. marneffei*. A continuous updating of current commercial databases provided by MALDI-TOF MS vendors together with the instrumentation as well as building up new databases for specialized research purposes is needed. MALDI-TOF MS is suitable for the routine identification of filamentous fungi in a clinical microbiology laboratory.

## Conflict of Interest Statement

The authors declare that the research was conducted in the absence of any commercial or financial relationships that could be construed as a potential conflict of interest.
